# Wargaming for Aircraft Manufacturer Product and Industrial Strategy

**DOI:** 10.1177/10468781251401426

**Published:** 2026-01-13

**Authors:** Fanny Camelia, Jeremy Smith, Ian Marr, Chris Lynas, Timothy L. J. Ferris, Ramona Dogea

**Affiliations:** 1 2717 Cranfield University, UK; 2 41755 Airbus Operations Limited, UK

**Keywords:** wargame, wargaming framework, role-playing, air transport systems, aircraft manufacturing plan

## Abstract

**Purpose:**

Air Transport Systems (ATS) are complex sociotechnical systems with dynamic stakeholder interactions and non-linear relationships. Traditional analytical planning methods often oversimplify these complexities, limiting decision-makers’ ability to understand system dynamics. This study introduces a wargaming framework, adapted from military practices, to help aircraft manufacturers align product development and business strategies with airline competition.

**Design/Methodology/Approach:**

Developed collaboratively by Cranfield University and Airbus UK, the framework consists of seven elements: objectives, players, game components, game mechanics, scenarios, facilitation and feedback. Its development followed structured engineering project stages design, preparation, testing and evaluation allowing for iterative refinements.

**Findings:**

The wargaming provides an interactive platform to simulate airline competition and stakeholder interactions, enhancing strategic decision-making. Validation exercises demonstrated its ability to model ATS dynamics, revealing insights into stakeholder behaviour, decision processes and competitive influences on aircraft development. The framework offers both epistemological benefits (deeper system understanding) and pedagogical benefits (improved strategic thinking and decision-making skills).

**Practical Implications:**

The framework serves as an analytical and educational tool, enabling aircraft manufacturers to assess market dynamics, stakeholder behaviours and competitive strategies in a structured environment.

**Originality/Value:**

This study presents a novel adaptation of wargaming approach that captures ATS complexities, supporting strategic planning for aircraft manufacturers. By positioning military-inspired wargaming within the broader game science literature, the study provides a methodological bridge between Design-in-the-Large (high level strategy) and Design-in-the-Small (specific game design elements), supporting future advancements in product and business strategy within ATS.

## Introduction

The Air Transport System (ATS) includes diverse and interdependent stakeholders, including passengers, airlines, airports, aircraft, aircraft manufacturers, and air transport networks (Nissalke, 2001; Schaar & Sherry, 2010; Spasojevic et al., 2018). This complex sociotechnical system involves social elements related to people and society, such as behaviours, attitudes, or preferences, and technical elements encompassing processes, tasks and technologies. The ATS is dynamic, meaning that any changes in its system elements significantly impact other system elements and the system as a whole. The system’s dynamic behaviour not only describes its evolution but also affects its function (Rocha, 2017). This can be observed from fundamental structural changes over the past few decades due to globalisation, deregulation and liberalisation as well as the introduction of Low-Cost Carriers (LCCs) (Kalemba & Campa-Planas, 2016; Widmann, 2016). These changes, driven by societal and technological trends, influence the values and expectations of stakeholders, resulting in dynamic behaviour (Beihoff et al., 2014), that continually reshapes the competitive dynamics within the ATS. These dynamics can lead to higher functionality of system elements, changes in emerging system properties, lower operational costs or lower customer prices, although they can also lead to increased costs, such as those associated with the introduction of carbon taxes, or volatility in fuel and energy supply markets.

One of the major challenges for aircraft manufacturers is understanding the complex and dynamic interaction among stakeholders within the ATS. A comprehensive understanding of complex stakeholder interactions, especially airline competition and strategies, and the implications that arise from these interactions, enables aircraft manufacturers to align their product development and industrial strategies with the evolving market needs. This is particularly essential in this capital-intensive industry that requires substantial investments in technology, engineering and manufacturing, and is characterised by long-term commitments and risk involvements (Reis et al., 2019; Schmitt & Gollnick, 2016). However, traditional analytical planning methods, such as linear programming for airlines’ fleet allocation, regression-based forecasting for aircraft demand, or discrete-event simulation for aircraft manufacturing planning, primarily relying on mathematical models and quantitative analysis, often fail to capture a holistic perspective of systems behaviours and interactions. These approaches tend to oversimplify reality and assume linear relationships, failing to fully capture the complex, non-linear and multi-layered interactions between stakeholders, including the social and behavioural aspects of human’s decision-making. Without a method enabled to simulate these interactions, decision-makers may not be able to fully realise the complexities of the system, its inherent dynamics and the implications of their decisions, leading to potentially suboptimal results.

To address this challenge, instead of using analytical planning techniques, this article proposes a wargaming-based framework as a structured method to model and simulate the real-world ATS’s scenarios. Situated within game science and inspired by military applications, wargaming is particularly suited to the ATS context because it captures competitive and hierarchical interactions, namely, competing airlines make strategic decisions on route planning and aircraft acquisition, and the manufacturer responds to these decisions, reflecting partially conflicting objectives among stakeholders. The framework involves key ATS elements including passengers, airports and routes with the primary exploration revolves around competition between airlines and the impact of these competitions to aircraft manufacturers. This emphasis is justified by the fact that airlines are the proximate customer of aircraft manufacturers and their fleet management and aircraft acquisition decisions which are shaped by passenger demand, market conditions, financial considerations, technological factors and environmental impacts (Khoo & Teoh, 2014) influence the aircraft manufacturer’s ability to anticipate demand and design competitive products. Moreover, given the limited literature on how the airline competition influences aircraft manufacturer planning, the study contributes to the literature by proposing a novel approach that captures the complexities of airline competition to support strategic decision-making in the aircraft manufacturing industry.

This study links the aircraft manufacturer’s real-world challenge (aligning product and industrial strategy with the dynamics of airline competition) to the design of the wargaming itself. In line with Klabbers (2009, 2018), this reflects the relationship of ‘Design-in-the-Large’, the broader strategic, social and organisational problem context and ‘Design-in-the-Small’, the specific game design elements that implement and operationalise this context in a playable format.

This article presents the development of a wargaming framework designed to support product development and industrial strategy from the aircraft manufacturer’s perspective in the ATS. It presents a literature review of wargaming, the components of the wargaming framework, its design and implementation, followed by a discussion on the reflections of the two iterations of wargaming design and validation. Finally, a conclusion summarises the key insights and implications derived from the study.

## Wargaming as a Method: A Literature Review

A wargaming, as defined by Perla and McGrady, leading defence wargaming experts, is “a warfare model or simulation, using rules, data and procedures, not involving actual military forces, and in which the flow of events is affected by, and in turn affects, decisions made during those events by players representing the opposite sides” (Perla, 1990). Traditionally rooted in military applications, wargaming was adopted by the Prussian military in the 1820s as a method to respond to the growing complexity and uncertainty of warfare, as well as the expansion of armies and the rise of industrialisation (Ministry of Defence, 2017; Sabin, 2014; Schwarz, 2013). During World War 1 and World War 2, it was used for war simulation, enabling the development of battle plans at both the tactical and strategic levels (Li et al., 2013; Peng et al., 2008; Zhen-Jiang et al., 2017). It was also employed during the Gulf War of 1991 to support military planning and warfighting, including exploring decisions about combat and logistics options (Mason, 2018). The military has utilised wargaming to understand the complexities of war and conflicts and to anticipate opponents’ decisions related to threats, opportunities and challenges (Goria, 2012; West et al., 2018; Wojtowicz, 2020).

Beyond the military, wargaming has been successfully adapted in various disciplines, across socio-technical systems, to simulate complex and dynamic interactions between different actors in a controlled environment. This includes the applications in the cyber domain, as highlighted in the works of Bumanglag et al. (2019), and Ormrod and Scott (2019) (Bumanglag et al., 2019; Ormrod & Scott, 2019); in education, as seen in the studies conducted by Reynaud and Northcote (2015) and Schwarz (2013) (Reynaud & Northcote, 2015; Schwarz, 2013); in emergency management, as evidenced by the research conducted by Chen et al. (2018) and Yu et al. (2015) (Chen et al., 2018; Yu et al., 2015); and in the field of public policy, with studies conducted by Oberholtzer et al. (2019) and Wojtowicz (2020) (Oberholtzer et al., 2019; Wojtowicz, 2020).

Wargaming has also been utilised in the business or corporate context, commonly referred as business wargaming, to support strategic decision-making, competitive analysis and market foresight (Gilad, 2008; Goria, 2012; Schwarz, 2009; West et al., 2018). For example, Kurtz (2003) utilised it to help an information technology (IT) company’s top management identify the best strategy for improving their market share. The wargaming aimed to explore the dynamic aspects of competition, identify stronger and weaker competitors and test the possible strategies. It enabled advising the company to avoid attacking stronger competitors and instead focus on taking business from weaker competitors, helping the company survived and became stronger (Kurtz, 2003). Other studies focused on developing a wargaming to enable foresight and improve a company’s foresight ability (Schwarz, 2009; Schwarz et al., 2019), to reveal insights on marketing dynamics in order to defend or take control of a particular market (Goria, 2012), to provide hands-on strategy training in management classes (Schwarz, 2013) and to support scenario development through a simulated real-life case study (Schwarz et al., 2019).

Wargaming has been proven to be effective for developing strategies and optimising planning between different stakeholders under uncertainty and incomplete information (Brown, 2019; McCreight, 2013; Ormrod & Scott, 2019), demonstrating the epistemological foundation of wargaming. It enables the generation of knowledge and the revelation of underlying dynamics and relationships, allowing decision-makers to anticipate emergent processes, reactions, counterreactions and future states, and to consider strategies and opponents’ choices, through its iterative cycles, particularly in situations where traditional analytical planning methods are unable to capture these dynamics and emergent behaviours. The research conducted by Jensen and Banks (2018) demonstrated analytical benefits of wargaming by demonstrating its capacity to isolate frequently recurring postures and preferences in conflict situations. They conclude that wargaming was a useful methodology for exposing likely preferences in cyber operations (Jensen & Banks, 2018). Furthermore, Heckman et al. (2013) reported a cyber intrusion wargame that allows the exploration of dynamic aspects through four teams. One of these teams, the attacking Red Team, was responsible for one perspective on the system. This study employed various attack and defence methods to assess the effectiveness of different strategies, demonstrating how wargaming can produce actionable knowledge to enable evaluation of strategies in the context of cyber intrusions (Heckman et al., 2013).

Wargaming is also an important method for training and education, emphasising its pedagogical foundation. Perla and McGrady (2011) emphasised that the value of wargaming lies in its ability to deeply engage participants and influence their thinking, feelings and behaviours. They argued that wargaming provides a unique and immersive learning experience that is more impactful than traditional forms of education. Wargaming provides educational benefits by promoting active learning and critical thinking as well as facilitating experiential learning in navigating complex and dynamic environments (Perla & McGrady, 2011). For example, Chen et al. (2018) discussed the implementation of wargaming focused on the complexity of urban flooding emergency rescue. This highlights educational benefits by providing a realistic simulation environment for training purposes on rescue operations during urban flooding (Chen et al., 2018). Similarly, Dorton et al. (2017) utilised wargaming to elicit diverse stakeholder perspectives during design thinking workshops. They showcased educational benefits of wargaming by facilitating a training for comprehensive understanding of stakeholder interactions and design options (Dorton et al., 2017).

Despite its strengths, some studies acknowledge the ongoing challenges of wargaming. Curry (2020) evaluated the application and practice of professional wargames, examines their historical accuracy and identifies errors to improve future game design, especially in geopolitical simulations vital for informing state policies and training decision-makers (Curry, 2020). Downes-Martin (2013) explored the challenges and complexities associated with adjudication in war games, particularly when facing new operational or strategic scenarios. The author explored the role of adjudicators, the potential risks of overreliance on game decisions, and the importance of understanding the dynamics between players, adjudicators and the control cell in order to draw reliable conclusions from wargaming exercises (Downes-Martin, 2013). Furthermore, a critical examination of the state of wargaming within the Department of Defence (DoD) and a call for more analytically rigorous approaches to wargaming was published by Compton (2019) and it reflected on the challenges and shortcomings in current wargaming practices, highlighting the need for improvement and greater integration with traditional analytical methods (Compton, 2019). Understanding these challenges is important for ensuring the utility and credibility of wargaming as a method for analysis and decision making as well as for education.

In the context of the ATS, wargaming provides an opportunity for aircraft manufacturers to explore the airline competition dynamics that traditional analytical planning methods cannot fully capture, to help them planning the strategic responses in terms of product development and industrial strategies. Previous studies, as discussed above, have applied wargaming in military, business and socio-technical contexts, however, its use to model both competitive interactions between airlines and their interplay with aircraft manufacturers remains limited. Therefore, this study adapts wargaming principles to simulate these interactions for supporting strategic decision-making, reflecting real-world ATS dynamics.

## Methodology: Wargaming Development

**Figure 1.**
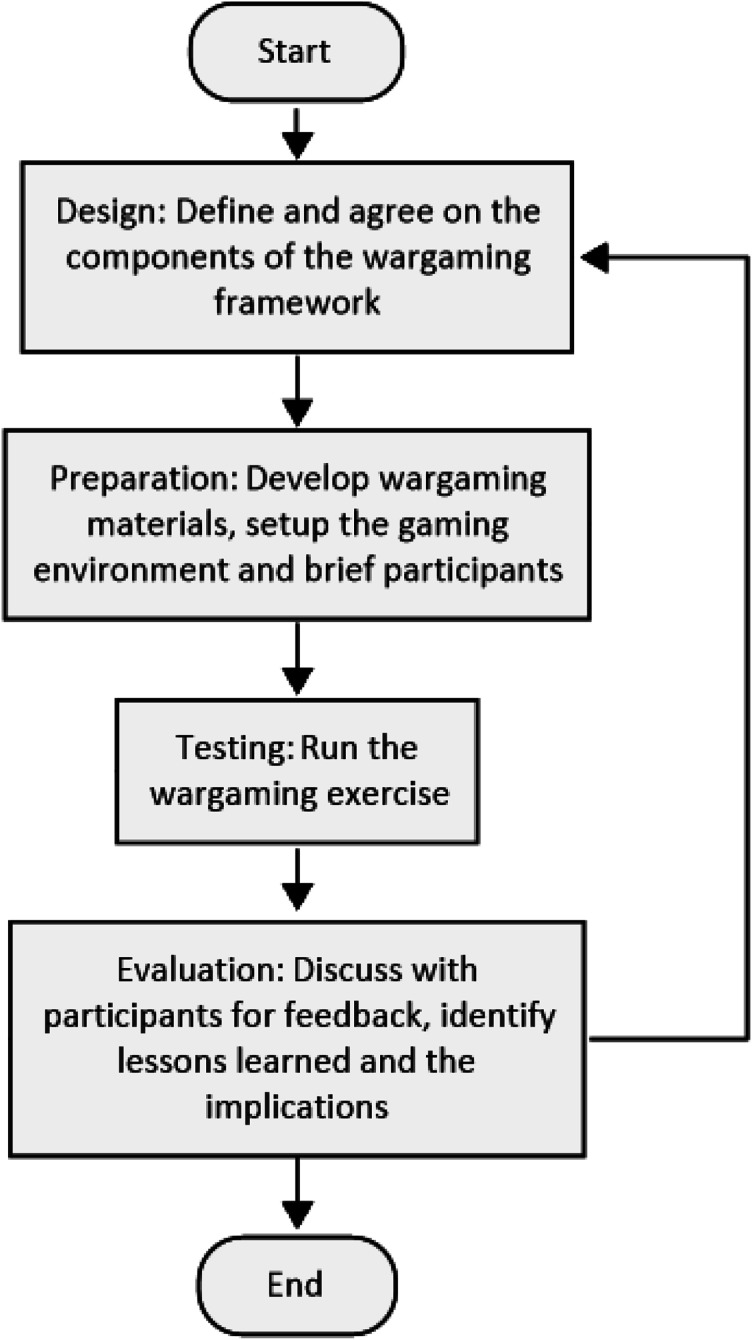
Wargaming development stages.

The wargaming framework, was co-developed by teams from Cranfield University and Airbus UK, an aircraft Original Equipment Manufacturer (OEM). The development and validation of this framework followed the structured and simplified approach outlined by Goria (2012) consisting of design, preparation, testing and evaluation (see Figure 1). This approach provides a structured and systematic process, linking Design-in-the-Large, reflecting the broader ATS organisational and competitive context, with Design-in-the-Small, encompassing the game design elements that operationalise this context into a playable simulation. This means that the game components, including rules and scenarios, are designed based on the real-world problem. Although this approach is simpler, it shares significant similarities with the more comprehensive methodology proposed by Kurtz (2003).

Table 1 outlines the key stages of Goria’s approach used in this wargaming framework development and highlighting how they align with proposed by Kurtz (2003). Goria’s approach, although more streamlined, also aligns with the typical stages found in development projects, investigation and preparation, execution, and post-project evaluation. An adjustment is made here, where the execution was replaced with testing because the output of this process is a system prototype rather than the final product of the wargame.

**Table 1.** Wargaming Development Stages Comparison.

Goria (2012)	Kurtz (2003)	Notes
Design	Definition of scope, design meeting	The definition of scope and design meeting steps in Kurtz’s approach are combined into the design stage in Goria’s approach
Preparation	Preparation	Both approaches include a preparation stage to set up the necessary elements for the wargaming exercise
Testing	Pre-wargame briefing, execution	Pre-wargame briefing and execution in Kurtz’s methodology are combined into the testing stage in Goria’s approach
Evaluation	Post-wargame documentation, debriefing and future planning	Post-wargame documentation, debriefing and future development planning are integrated into the evaluation stage in Goria’s approach

### Design

The wargaming framework was developed during the design stage. The framework includes key components including objectives, players, game elements such as maps and counters, rules or game mechanics, scenarios, facilitation and feedback and evaluation that reflect real-world challenges. This stage consisted of a series of meetings centred on knowledge exchange workshops aimed at collaboratively designing the wargaming framework for supporting planning and decision-making in the development and manufacturing of aircraft. During the workshops, as the nature of knowledge exchange, the Cranfield University team shared their knowledge about the theoretical aspects of wargaming and their previous experiences in developing military wargames. Simultaneously, the Airbus UK team shared knowledge about the situation and the main issues faced by aircraft manufacturers, particularly in aircraft product development and industrial strategy planning. Collaboratively, the components of the wargaming framework were defined and agreed upon, translating the broader ATS context (Design-in-the-Large) into playable game design elements (Design-in-the-Small) (Klabbers, 2009, 2018).

### Preparation

The preparation stage consisted of collaborative efforts to ready the game for testing, involving the development of game artefacts, setting up the gaming environment, preparing the brief for participants. During this stage, the team prepared physical game elements including maps and counters, developed and automated the computational aspects of the wargame such as finance calculation and created informative documents detailing the wargaming framework to be shared with prospective testing participants.

### Testing and Evaluation

The subsequent stage focused on the validation of the wargaming framework through testing and evaluation activities. Testing was conducted through a wargaming exercise aimed at testing, reviewing and validating the wargaming framework involving participants from both the Cranfield University and the Airbus UK. The wargaming exercise was conducted as a one-day workshop in July 2019, involving 15 participants split into three teams and a facilitator team. Three players were assigned to each team: an airline (Blue Team), a competing airline (Red Team), and an aircraft manufacturing company (Green Team). It also involved a facilitator team consisting of an umpire and five control staff to guide the game, provide information on market conditions, update the calculation sheets and ensure smooth gameplay. The exercise was concluded with a debriefing session where all participants reflected on their experiences, shared their observations and provided verbal feedback. This qualitative feedback was collected to identify emergent insights, lessons learned and potential areas for further iterations.

### Second Iteration of Wargaming Development

Due to funding limitations, the second iteration of wargaming development was held much later. This followed similar stages as the first iteration, design, preparation, testing and evaluation, except the design stage was more streamlined, as the basic elements had already been developed during the first iteration. While the first iteration involved three workshops, the second design iteration was more efficient, requiring only one focused design meeting. Some improvements were made on the wargaming framework based on the feedback from the previous iteration. These improvements included changing the map to a larger size, automating more calculations to support game metrics and visualisation, and refining the rules and scenarios. Specifically, in the first iteration of development, only revenue calculations for airlines were considered, based on the revenue generated by allocating aircraft to routes. Meanwhile, in the second iteration, more sophisticated automated calculations and visualisations were developed. The second testing event was conducted as a one-day workshop in May 2023 and involved a larger number of participants, 25 in total. Eight players were assigned to competing airlines, Blue Team and Red Team, and three players to the aircraft manufacturer (Green Team), mostly a different set from those in the first iteration. Similar to the first testing, there was also an umpire and five control staff, who largely remained the same as in the first iteration. Like the first wargaming exercise, this testing also concluded with a debriefing session, where participants provided qualitative feedback to identify emergent insights and lessons learned and inform potential refinement for further development.

## Wargaming Framework: Design, Components and Implementation

**Figure 2.**
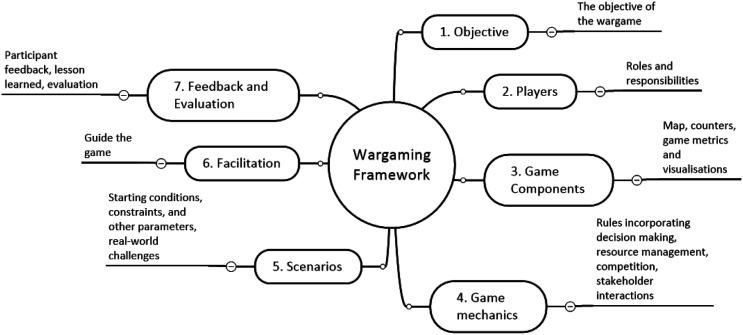
Wargaming framework.

The wargaming framework developed and refined through two iterations of development discussed in Section 3 consists of seven components designed to create a dynamic and immersive simulation. Each game components (see Figure 2) is deliberately linked to real-world challenges within the ATS, bridging Design-in-the-Large with Design-in-the-Small. This holistic framework enables participants to explore its complexities on airline competition and its impact on aircraft manufacturer decision making.

### Objective

The objective helps to create a coherent and integrated wargame that provides direction and focus for achieving specific goals. The primary aim of the wargaming is to simulate complex stakeholder interactions within the ATS, including dynamic competition among airlines, and their implications for aircraft manufacturers’ product and industrial planning. This objective is aligned with the aircraft manufacturer’s challenge in anticipating airlines’ behaviour and aligning long-term product and industrial decisions with a competitive ATS environment. The wargaming framework provides a structured yet flexible environment where airline strategies regarding route expansion and aircraft acquisitions strategies as well as the manufacture’s planning and strategies could be explored.

### Players

In a typical wargaming, there are usually two rival team players, with the Blue Team representing the friendly forces and the Red Team representing the enemy. However, more teams may be involved in the wargaming. In this study, the wargaming simulates two levels of interactions, competition between airlines and interactions between airlines and the aircraft manufacturer. Therefore, it involves competing airline teams (Red and Blue, or potentially more) and a manufacturer team (Green, or additional teams if needed).

The first set of players take on the roles of airlines, and these teams decide their approach to serving customer demand by allocating aircraft in their inventory to routes. Airlines make purchase orders to manufacturers to expand routes and generate revenue, engaging in negotiations regarding price and delivery time. They are positioned in a competitive relationship (Red vs. Blue teams) to reflect real-world competitive dynamics as they compete for dominance and market share in specific ATS networks. This aligns with Sun et al. (2024) study which highlights that network competition and route entry/exit decisions within the network are key aspects shaping airline competition (Sun et al., 2024).

The other set of players are manufacturers. The aircraft manufacturer (Green team) sells aircraft manufactured in their Final Assembly Lines (FAL) to airlines and reinvest revenue in additional FALs or in development of new and upgraded aircraft development programmes. These participants decide on the development of aircraft linked to the aircraft’s operating capabilities and align product and industrial strategies with market demands.

All players, similar to real-world businesses, made real-time decisions to sustain their businesses financially within the simulated environment, aiming for long-term survival and financial growth. Interaction among players, both within the team or across the teams enabled engagement and collaboration and fostered teamwork, communication and consensus-building skills.

### Game Components

Various game components, including maps, counters, and evaluation metrics, in this case supported by revenue calculations, were developed to help players contextualise and understand various concepts and relationships, providing a realistic and immersive simulation environment for ATS stakeholder interactions.

#### Map

The wargaming map depicts areas where the situations in question (often wars or conflicts) occur (Tan et al., 2010). There are three types of wargaming maps, namely, geomorphic or hexagonal grid maps, geographical (or landscape) maps and point-to-point maps (Goria, 2012). The point-to-point map is the most suitable for this wargaming exercise due to its ability to abstractly portray the link between airports through flight routes in an ATS network.

**Figure 3.**
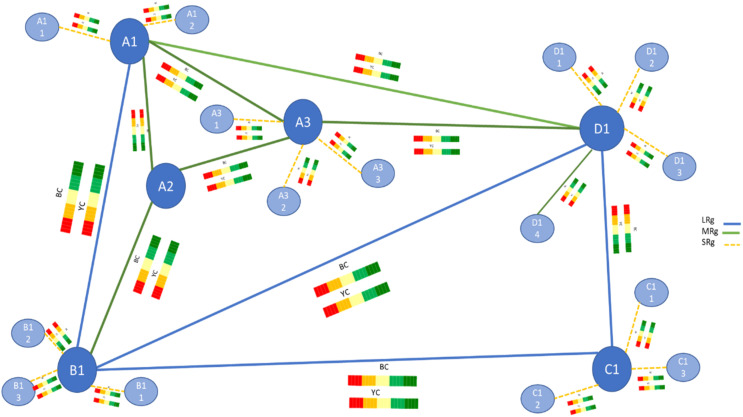
Wargaming map.

The map, as shown in Figure 3, depicts a hypothetical air transport network, with airports and routes represented by nodes and edges, to capture the key airlines’ competitive dynamics. It provides a structured environment where airlines’ strategic decisions on route planning could be observed and examined. Although the map does not scale distances to real-world measurements as it is entirely hypothetical, it differentiates distances into long, medium and short ranges. This differentiation helps players perceive the map space as a relevant feature by reflecting the relative distances and connectivity between different airports that can be served by long, medium and short-range aircraft. Passenger demand is also shown on the map. These elements, airports, routes and passenger demand, are explained below.

##### Airport

Airports are differentiated into major and secondary airports on the map, represented by dark blue circles and light blue circles, respectively. The major airports in the map (A1-D1) represent high-capacity airports that concentrate traffic and provide opportunities for hub-and-spoke strategies for airlines. Meanwhile, secondary airports (such as A11, A12) represent low-capacity airports to represent the growing role or regional and low cost carrier (Dobruszkes et al., 2017; Forsyth, 2010). This reflects the real-world challenge of operational constraints for route planning as airlines typically operate in both types of airports to dominate an ATS network. Airport capacity, however, can restrict the addition of flights on a particular route.

##### Route

The route connecting airports is characterised by the typical range of aircraft needed to operate on it, differentiated as long-range, medium-range and short-range. Each route is also characterised by the cost of operation and revenue per passenger in each seating class. This reflects the real-world challenge airlines face when allocating aircraft to routes, balancing financial considerations, passenger demand and pressure to win market share.

##### Passenger Demand

To factor passenger demand into the game and represent market considerations so that airlines can respond to varying demand, the map exhibits each route’s demand by using the rainbow marker track system. The rainbow marker track system was adapted from the previously developed military campaign wargaming to describe the level of support and opposition of the population to military operations (Smith & Barker, 2022). In this study, similar approach was adopted to represent the level of passenger demand and demand fluctuations. It also shows the varying levels of demand that have currently been met by airlines as both Red Team and Blue Team can serve passenger demand on each route by allocating their aircrafts.

**Figure 4.**
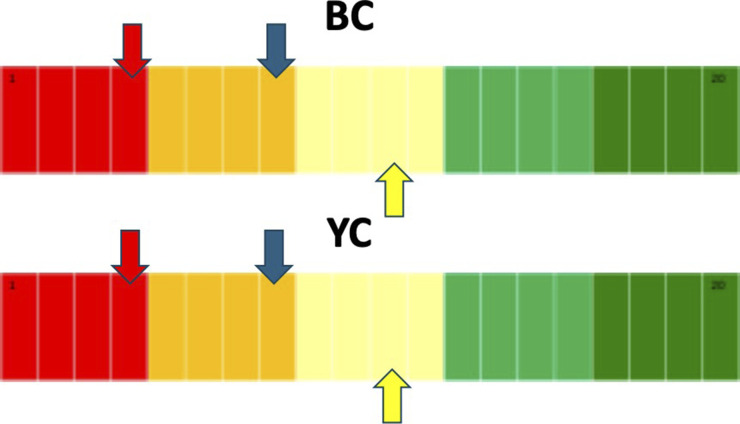
Rainbow marker track system.

Each marker has a scale that runs from 1 (minimum number of passenger demand) to 20 (maximum number of passenger demand). Separate markers can be used for business and economy classes (BC and YC, respectively). An example of the use of markers is shown in Figure 4. The yellow marker indicates the level of passenger demand (level 11). Meanwhile, the red marker indicates that the Red Team has accommodated a lower level of passenger demand (level 4) than the Blue Team (level 8), represented by the blue marker. The rainbow marker track system in Figure 4 suggests that more passenger demand could be accommodated so that the Blue or Red Team could allocate more aircraft on this route.

#### Counters

Counters provide important information for the wargame (Goria, 2012). These tangible physical elements help to visualise concepts and interactions. In military wargames, for example, land, naval and air units are represented by counters. Each counter could have its properties, for example, the strength, size or movement allowance for military units. The counter sets developed for this wargame are explained below.

##### Airport Grids

**Figure 5.**
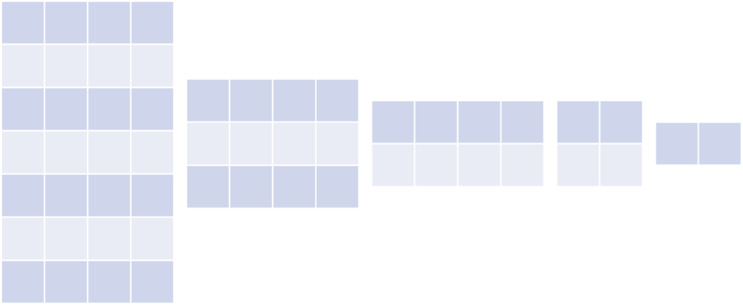
Airport grid system examples.

Each airport, represented by the blue circles on the map (see Figure 3), is assigned a counter to indicate operational capacity. The capacity, represented by the grid system which shows the number of available slots at each airport, enables participants to explore how airport capacity affects route planning, airline competition and the manufacturer’s strategic responses. Figure 5 shows airport grids of various capacities, sequentially from the largest to the smallest capacity. Small counters or notes can be added to the grids as the aircrafts are assigned to routes to and from the airport, representing the capacity (Figure 5). The capacity restricts the number of aircraft that can operate on a particular route, therefore, once all slots at a given airport are filled, no more aircraft could be added to routes connecting to that airport.

##### Aircraft Units

Aircraft units are the main counterset in this wargame. This included a variety of aircraft units with different capabilities and costs, representing the airline’s fleet for both the Red Team and Blue Team. Specifically, the aircraft is characterised by the following elements.

(1) Capacity for both business and economy class
(2) Range, including long-range, medium-range, and short-range
(3) Baseline efficiency, the operational efficiency reduced through the life cycle
(4) Slot coverage requirement in the airport (the number of slots this aircraft type takes on the grids for the airports)

**Figure 6.**
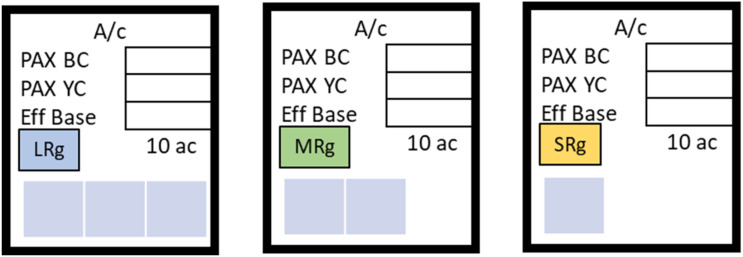
Aircraft units.

Example aircraft counters are shown in Figure 6. Each counter represents a number of aircraft (a default is 10, but this may be varied). In this example, the counter has space for passenger carrying capacity in the two classes (business and economy) and aircraft efficiency. It also specifies aircraft range and the number of required airport slots, as grey squares correspond with the grid system for the airports. A Long Range (LRg) aircraft requires three airport slots, a Medium Range (MRg) aircraft requires two, and a Short Range (SRg) aircraft requires one (see Figure 6), reflecting operational constraints that airlines must consider when allocating aircraft resource on particular route. This allows airlines to explore trade-offs between aircraft range, airport capacity and competitive positioning, while also observing how their choices influence aircraft manufacturing strategic decisions.

##### Revenue and Range

**Figure 7.**
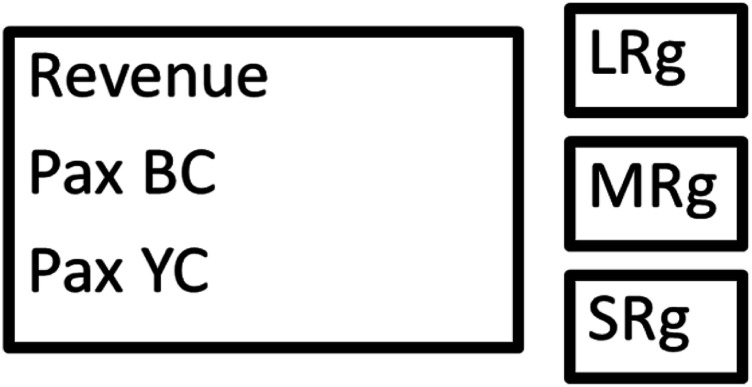
Revenue and range counter.

The revenue counter provides revenue information that airlines can earn by allocating their aircraft on specific routes, enable players to explore trade-offs between profitability of the route and aircraft characteristics, supporting strategic decisions on fleet allocation. Meanwhile, the range counter informs the aircraft type needed for a particular route. Both are to be placed on each route during the wargaming exercise. Figure 7 illustrates the revenue and range counter.

##### Final Assembly Line (FAL) Scheduler

**Figure 8.**
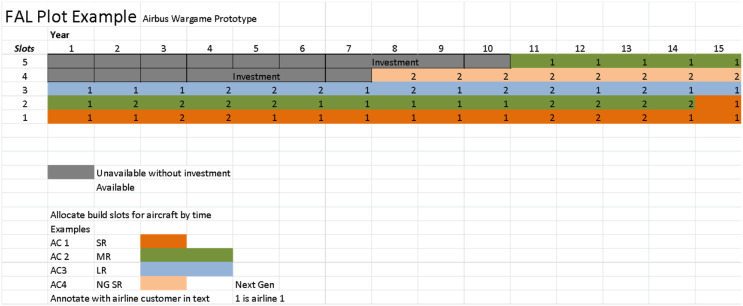
Final assembly line (FAL) scheduler.

The aircraft manufacturer has a Final Assembly Line (FAL) scheduler table/plot in the form of a grid (see Figure 8 below). This grid shows the assembly slots that allow manufacturers to allocate slots for building new aircraft. When ordered by airlines, the aircraft join the scheduling queue on the FAL for the corresponding aircraft type. Orders move along the FAL at one square per turn until delivery. This grid requires the manufacturer to allocate builds by year and by line and to allocate slots for the builds of new aircraft. It enables participants to explore the impact of airline orders on production planning.

##### Counter Size

The dimensions of the counters in the game are proportional to the size of the map, to provide players with a quick visual perception of what each counter represents, and to facilitate ease of movement and manipulation during gameplay. Counter sizes vary based on their symbolic significance and manipulative convenience within the game. For example, the airport grid counter size depends on the airport’s capacity with larger airports have bigger counters, reflecting their higher capacity. The aircraft unit size is approximately 25 mm, allowing for easy handling and movement. Revenue and range counters are sized around 100mm x 60mm, while the FAL grid counters are about 300mm x 200mm, ensuring they are visible and distinct from other counters while remaining manageable for participants.

#### Automated Calculations and Visualisations

Depending on the level of automation, wargaming can be classified into manual wargaming, computer-assisted wargaming and fully automated wargaming (Hodický et al., 2020). Manual or seminar-style wargaming does not involve computers and are dominated by human-centred activities. Computer-assisted wargaming automates specific elements that involve human activities, such as conducting analysis, predicting outcomes, or making tactical decisions. Fully automated wargames, on the other hand, replace all human roles, although such systems are not always regarded as wargames per se (Hodický et al., 2020). Despite the growing interest in implementing emerging AI technologies into wargaming, manual wargaming is still much in demand as they are particularly well-configured to encourage and allow participants to interact and to experience uncertainty and dynamic scenarios. Manual wargaming is also practical, convenient, and cost-effective, with shorter development cycles, which is especially applicable to business wargaming and commercial timeframes.

**Figure 9.**
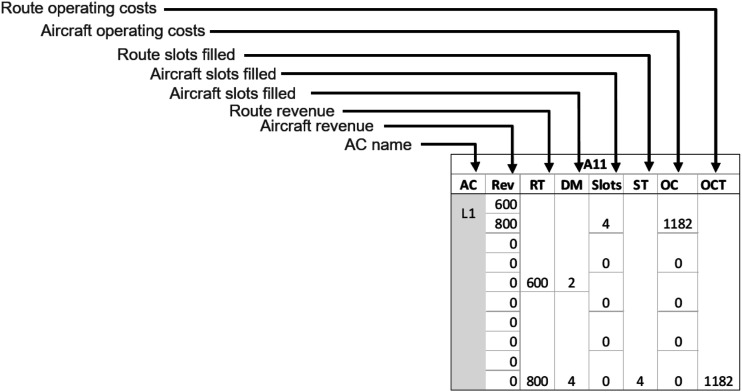
Airline finance calculation.

This study used automated elements, combined with manual wargaming, to support decision-centred tasks by providing aids such as progress bars, live revenue and cost tracking, fleet degradation and airport capacity calculators and templates. To provide a comprehensive understanding of airline financial positions, a macro-enabled Excel workbook consisting of several spreadsheets was designed to automate game calculations and provide real-time feedback to players while minimising manual input. The first spreadsheet stores data relevant to all players in the game, such as revenue per seat, operating cost per seat, route lengths, slot availability at airports, route demand and capacity, financial settings for airlines, and aircraft upgrade programme probability distribution settings for the aircraft manufacturer. These values are used for further calculations. Two other spreadsheets record the in-service aircraft owned by each airline (Red and Blue teams) and their characteristics, as well as calculating operational data (e.g., revenue, operating cost, filled airport slots, route demand). An example of the calculation is provided in Figure 9, demonstrating how to calculate revenue, route demand met, airport slots filled by the airline, and operating costs at the aircraft and route levels, representing airline decisions in quantifiable outcomes. This is part of the airport matrix, with rows and columns intersecting to represent the routes between them.

**Figure 10.**
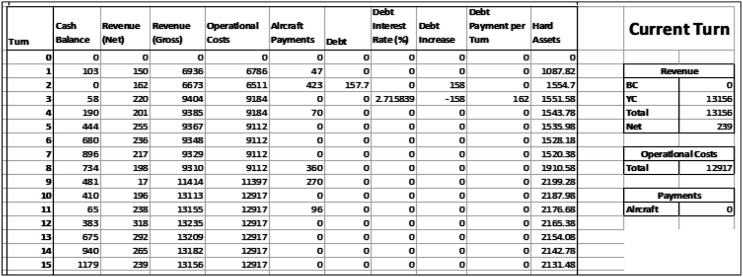
Airline finance summary.

Two additional spreadsheets summarise each airline’s financial position (e.g., overall revenue) and aircraft orders for each turn. Figure 10 provides examples of turn-by-turn airline finances, including cash balance, revenue, operational costs, aircraft payments, debt, debt payments and hard assets at each turn.

**Figure 11.**
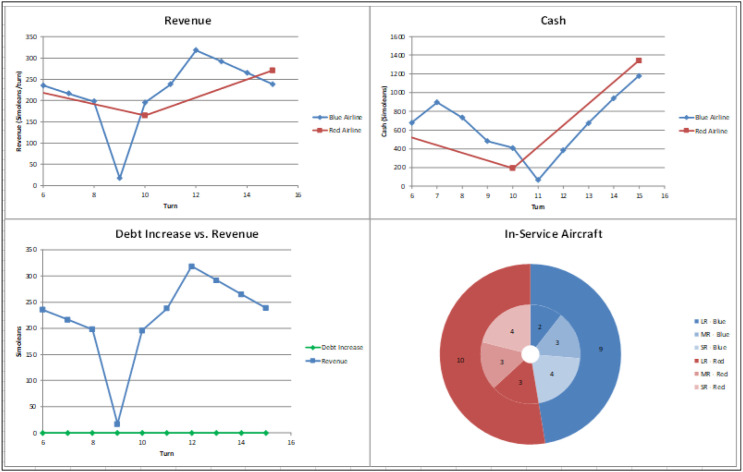
Airline performance visualisation.

The final two spreadsheets visualised the data, enabling airlines to make informed decisions quickly. Figure 11 shows an example of the visualisation of airline performance in terms of finance and in-service aircraft in the network during a particular round to inform both teams. These calculations and visualisations help participants to understand competitive dynamics between rival airlines and allow the aircraft manufacturer to anticipate the strategic responses.

### Game Mechanics

**Figure 12.**
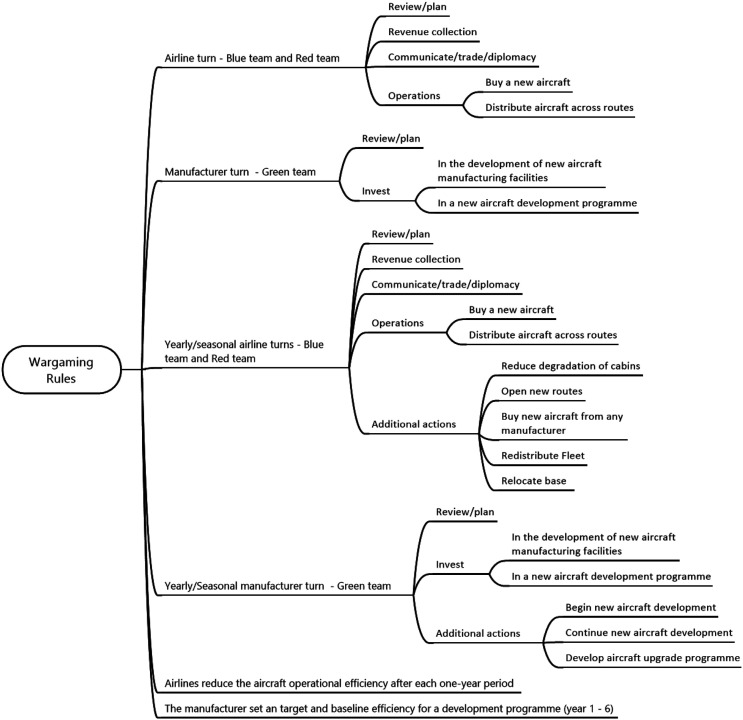
Summary of wargaming rules.

The game mechanics, or rules, provide structure and boundaries of the game by governing turns, movements, rounds, sequences of play and interaction between participants. They are designed to follow natural and physical laws and human behaviours (Li et al., 2013). Various aspects incorporate into game mechanics including competition, decision-making, resource management and stakeholder interactions. For example, turns represent time cycles, resource management is reflected by airport slots and FAL production scheduling and competitive moves illustrate airline rivalry for routes and market share. The rules for this wargame, played in both testing iterations, are provided in Figure 12.

To enable efficient exploration of competition, strategies and impacts, the game operates on a baseline turn system, where the initial turn duration is set to one month by default. However, the system is designed to allow jumps for longer periods where all participants agree that the situation is steady. The system is also designed to allow for flexible turning lengths. Turns can be flexed to be of any length up to a year. This flexible structure for duration of turns results in different granularity of the game, which enable more efficient exploration of the effect of particular class questions or the differences in decision-making timeframes typically observed between airline and aircraft manufacturer operations. This has to be done with the agreement of all participants.

Both airline and aircraft manufacturer have to make strategic decisions on aircraft purchases for airlines, and investments for aircraft manufacturers. For airlines, this means deciding on aircraft purchases and route strategies to balance passenger demand, revenue generation and competitive positioning. Each airline needs to perform some activities for each turn, including review/plan, revenue collection, communicate/trade/diplomacy and operations (see Figure 12). On the other hand, the manufacturer for each turn, conduct some activities, including review/plan and investment, making strategic decisions about aircraft development and market alignment. However, the game mechanics are a mix of strict protocols and flexible elements. Players are not limited to predefined moves and can propose any strategies or actions relevant to their roles. This allows creative participant actions that do not constrain possible actions by adhering too strictly to traditional practices in the field, reflecting the uncertainty and adaptability inherent in the ATS.

Each full year, airlines and manufacturers have additional possible actions related to resource management such as opening new routes and redistributing the fleet for airlines and developing aircraft upgrade or new aircraft programmes for manufacturers, which could also have been done seasonally within the year (see Figure 12). These annual and seasonal decision cycles mirror real-world industry practices and can encourage participants to consider trade-offs between short-term profitability and long-term survival and growth.

Interactions among stakeholders are explored to understand the implications of relationships on decisions within the ATS. Airlines’ communicate/trade/diplomacy activities could include discussions with airports if they are played (or with Control staff who can represent them), manufacturers, and also between the airlines. These interactions help to explore the implications of stakeholder relationships on decision-making within the ATS. Presumably, these players could be given scripted potential responses to scenarios based on the drives/motivations of the particular entity in the system; thus, different airports might follow different policy-related scripts, which could be predictable given the history and ownership. The alternative would be unscripted interactions where the players can decide freely in real-time.

After each simulated year, the aircraft’s operational efficiency reduced, reflecting the real-world effects of aging and wear. Therefore, the airline have to lower the efficiency of the aircraft, which could impact route profitability and consequently airline actions or decision-making. This degradation could be randomised or just set as a default or deterministic. Randomised or deterministic degradation serve as a means to reflect different bases of degradation. In principle, various events could cause aircraft degradation, but it is unknown which aircraft the event occurred to and how severe the effect is. The randomised approach indicates different rates of cumulative impact; meanwhile, the deterministic indicates that it all averages out at the airframe level per year.

In the game, the manufacturer has time and other constraints to complete an aircraft development programme to achieve aircraft performance improvements, which are costly but could provide efficiency benefits that made the product more desirable. This impacts decision-making for both airlines and aircraft manufacturers. For each programme, the manufacturer set an initial efficiency target for a development programme, for example, 120% and a baseline efficiency for accomplishing the aircraft development programme from year 1 to year 6. The manufacturer could have a randomised effect in finalising a development programme affecting the baseline efficiency each year. This reflects the uncertainty and risks inherent in new aircraft programme development that influence on both production planning and airline fleet decisions.

### Scenarios

**Figure 13.**
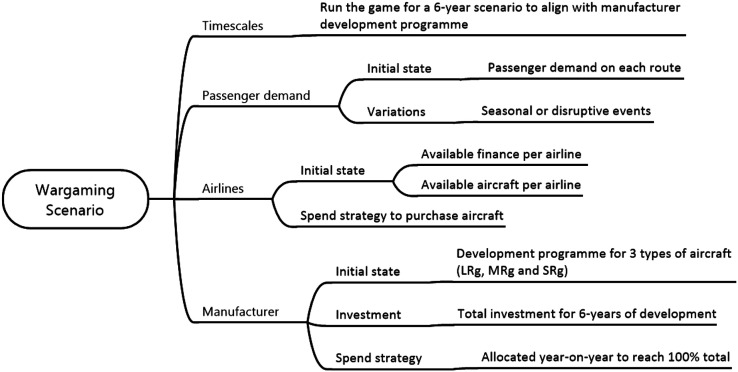
Example of wargaming scenario.

The wargame may be played against several scenarios. These scenarios reflect real-world challenges faced by airlines and aircraft manufacturers, providing context and narrative and facilitating experiential learning. Scenarios define starting conditions to represent resource constraints, such as an airline’s access to finance, which can significantly impact its ability to purchase new aircraft. They also define aspects such as available aircraft characteristics, airport capacity restrictions, seasonal variations, manufacturer lead times, unexpected disruptions and skewed market conditions, amongst other potential scenario options. All these may be varied. Example conditions for a scenario, played in the first testing iteration, are presented in Figure 13. Airlines are allocated a certain amount of financial resources at the beginning of the game, which they have to manage carefully to expand their fleets and operations. The game also includes unexpected events or market disruptions to test participants’ adaptability and decision-making skills, such as fluctuations in passenger demand during holiday sessions and disruptive events such as a pandemic that suddenly reduces passenger demand significantly over a period of time.

There may be a random effect on the efficiency degradation for aircraft at annual turns. In this example, a random number is generated with a 10-sided dice. The degradation scenario is determined as follows.

• If the dice roll is 1 (representing less than 10%), no degradation occurs.
• If the dice roll is 10 (representing greater than 90%), a 20% degradation occurs.
• For all other rolls (2–9), the aircraft experiences a default 10% degradation.

Therefore, most aircraft degrade by 10% each year, with a small chance of either no degradation or a more severe 20% degradation.

Random effects can also be incorporated in the new aircraft programme development efficiency to represent risks and uncertainty in achieving target efficiency. Similar to aircraft degradation, a random number could be generated with a 10-sided dice. Each year’s efficiency is based on a baseline target increment plus a probabilistic adjustment determined by the dice roll:

• Year 1: Target efficiency is 120% with a baseline of 70%.
  - Probability <10% results in a 5% increase (better than predicted).
  - Probability 11–50% results in no change (70%).
  - Probability 51–80% results in a 5% decrease (worse than predicted).
  - Probability 81–100% results in a 10% decrease (60%).
• Year 2: Baseline addition to Year 1 is 10%.
  - Probability <10% results in a 2% increase (better than expected).
  - Probability 11–70% results in no change (the baseline addition of 10% is applied as planned).
  - Probability 71–100% results in a 2% decrease (worse than expected).
• Year 3: Baseline addition to Year 2 is 8%; same probability rules as Year 2.
• Year 4: Baseline addition to Year 3 is 6%; same probability rules as Year 2.
• Years 5–6: Baseline addition to the previous year is 3% per year; same probability rules as Year 2.

Each year’s efficiency builds incrementally on the previous year, with small random variations that can slightly improve or reduce performance. Therefore, in general, the efficiency remains close to the baseline, with only occasional substantial variations.

### Facilitation

The wargaming requires an umpire or facilitator role, can be fulfilled by either a single person or a team. These individuals, also known as control staff, oversee facilitation, adjudication, moderation, and event control throughout the wargaming exercise to ensure the objectives are met. In this role, the facilitators analyse the events and activities in the game, determine outcomes and adjust the results according to algorithms, rules and their expertise (Li et al., 2013). The umpire, with the help of control staff, facilitates the games and is responsible for briefing the participants on the rules and scenario at the outset, managing turn rotations, communicating players decisions to all participants, seeking approval from all players to advance the game if the situation is stable, and updating the calculation and visualisation on each turn in the spreadsheet.

### Feedback and Evaluation

Feedback and evaluation are important for generating empirical insights into the effectiveness of the game, the lessons learned, and implications for future refinement of the wargaming framework. Qualitative feedback collected from participants during debriefing session after both wargaming exercises indicates that the game was not only fun and enjoyable but also very successful at engaging participants. Participants agreed that the wargaming is an effective method for exploring ideas, as well as for identifying airline strategies regarding aircraft purchasing and manufacturers’ strategies for planning aircraft development programmes. The structured evaluation provided actionable insights and recommendations for further refinement of the game, some of which were implemented in the second iteration, while others are planned for future refinements.

## Discussion: Reflections on Two Iterations of Wargaming Design and Validation

This wargaming framework consists of seven basic elements, i.e., objectives, players, game components, game mechanics, scenarios, facilitation and feedback and evaluation. It was developed by translating the real-world problem context of aligning aircraft manufacturers’ product and industrial strategies with airline competition (Design-in-the-Large) into specific game structure and elements (Design-in-the-Small). The framework was designed, developed and validated following two iterations of a simplified process similar to a typical development project, including design, preparation, testing and evaluation. This provided a structured approach to ensure the framework was aligned with objectives and enables continuous improvement. The discussion in this section is based on qualitative feedback from participants collected during the final stage of evaluation, reflecting on the two iterations of wargaming design and validation.

### Objective

Although the objective was delivered quite clearly during the first testing, the participants in the second testing reported that they did not understand the objective of the wargame. Some participants felt uncertain about the purpose of playing as the objectives of game was not made clear at the start. Although a good understanding developed by the end of the day, nevertheless, some players felt lost during the game. Reflecting on this, objective definition is considered one of the most important aspects of the wargaming framework that needs to be delivered clearly to players before the game to ensure the game can achieve its intended outcomes.

### Players

In the first wargaming exercise, the number of players in each team was relatively smaller (3 players for each airline and aircraft manufacturer team), meanwhile, in the second test, they were larger (8 players for each airline and 3 players for the aircraft manufacturer team). However, the increase in team size for airlines in the second game was found to hinder effective communication, collaboration and teamwork. Therefore, it is recommended to maintain smaller team sizes, 3-4 players each team, to facilitate more effective coordination and decision-making among participants.

The current wargaming includes two competing airlines, which can also be viewed as consisting of the airlines of interest and rest of the industry, with the scaling of those airline purchases to represent ‘phantom’ airlines do not present in the main game. However, future iterations could extend the wargaming environments to represent more than two airlines by involving airlines of interest and other stakeholders in the environment.

Airports, for example, could also be represented by players if they can make decisions that contribute to the outcome. Adding players from airports would allow airlines to engage in negotiations, such as increasing capacity at an airport. This approach is common for business wargaming, as is the emphasis on role-playing, enabling the participation of different stakeholder roles (Kurtz, 2003). Adding players from competing manufacturers also could be a very useful augmentation, reflecting the current real situation where multiple manufacturers compete for market share. This would allow airlines to have the option to buy aircraft from others, and the manufacturer to have opponents to test their development strategies against their competitors. These additional players would balance the game and provide richer insights into two levels of dynamic competitions, although it would make negotiations much more complex and would require more careful facilitation.

### Game Components

Participants in the first iteration suggested making the map and counters bigger to allow writing/features to be more visible. These suggestions were implemented in the second iteration, where the map size was increased from 55.9 x 86.4 cm (A1 page format) to 300 x 200 cm. However, it is important to note that the map size should be tailored to accommodate the number of participants who will surround it and the number of counters to be placed on it.

Although it looks simply, the map, however, was perceived as complex when run in the game. This emphasises the importance of simplicity as the key to wargaming. Schuurman (2017), who reviewed different types and functions of wargames developed between 1770 and 1830, suggesting that simplicity is the most important requirement for wargaming playability. He further suggests that the wargaming designers must consider trade-offs between realism and playability (Schuurman, 2017). This implies that although some recommendations about additional players, additional rules, or additional scenarios appear realistic, they may increase the complexity of the game, thus, in turn, reducing the playability of the game. More assessments in the future need to be done to ensure that the game remains playable with additional elements of the game. This might include an exploration into thresholds related to playability and the conclusions that can be drawn from complex scenarios, such as the use of aircraft across multiple routes rather than being dedicated to specific routes.

To compensate for the complexity of the current network in the map, participants recommended automating as many of the calculations as possible. This would reduce the manual overhead associated with the current network and administrative burden placed on participants, enabling greater focus on human decision-making elements and more efficient use of the existing map whilst retaining the representativeness of the complex route network dependencies observed in real life. Therefore, while in the first game the automated calculations were minimal (only revenue for each airline), more calculations were automated in the second game, including cost, investment, and depreciation calculations, aircraft condition effects and airport/route capacity status updates. In addition, during the second game, the central revenue calculator and visualisations were live and displayed to all participants throughout the game so that all players knew their financial position round by round, although the visibility of their competitor’s financial position was only available at limited intervals to represent typical market reporting traits.

### Game Mechanics

This wargaming mechanics were deliberately designed to be flexible rather than all-encompassing that requires realistic professional approaches by participants, supported by expert control staff to ensure that it leads to reasonable and useful outputs. Unlike recreational games where rigid rules underpin and enforce the play, it is a framework for generating realistic issues and problems that can be studied and analysed within flexible game mechanics. These flexible elements enabled a structured yet adaptive gameplay experience to maximise its effectiveness. Modification of the rules, either between games or even at certain times within games, could be done to enable the exploration of different scenarios. The facilitator played the critical role is to ensure that the rules for a particular game iteration are followed so that the game’s results can be interpreted to inform decisions about how to respond to the environmental scenario changes represented by the changes in game rules.

### Scenario

It is notable that determining the most appropriate calibrated parameter values, for example, for the initial financing for each airline to be used in wargame scenarios, is difficult, especially when the game is tested for the first time. Wargaming exercises demonstrated how sensitive the outcomes were to the initial state; therefore, it is important to provide representatively scaled figures for the initial financing of each airline. Participants noted that initial number of aircraft owned by the airline as the initial figures were considered high, and in these conditions the airline is able to purchase aircraft at an unrealistic rate compared to the size of the existing network. Careful calibration to the initial setup is therefore important to fine-tune and enforce specific scenarios, such as block-booking of production slots by cash-rich airlines. Further research and calibration are needed to estimate the best configuration values. Additional tests may be useful for ensuring that the figures are reasonable and playable in the game. In addition, a list of random events may be needed to represent transient effects, observed variants and disruptions that can be introduced into scenarios to test the robustness of the strategies chosen by airlines and manufacturers under different conditions, for example, the introduction of financial, policy, operational, environmental or technological disruptions.

### Facilitation

The facilitator played a significant role in this wargaming, as they informed, steered, regulated, paced and arbitrated the game throughout. In this study, facilitation proved important to ensure a smooth gameplay experience. To enhance the facilitation, clearer instructions and rules was identified as an area for improvement. Communicating technical and financial assumptions such as scaling for aircraft seat capacity and cost scaling, would also help avoid confusion among participants. This highlights that importance of developing user guides and communication/assumption packs to be provided to participants before the exercise.

### Feedback and Evaluation

Debriefing sessions held at the end of each gaming session were critical aspect of the wargaming framework as they provided systematic opportunities to capture participants’ insights and reflections on strengths and weaknesses of the game. These sessions also identified opportunities for improvement, including refining the game mechanics and scenarios for future iterations, and securing buy-in for any proposed enhancements. Furthermore, the sessions enabled participants to share knowledge and insights, therefore, improved collaborative learning and the overall gaming and learning experience. Debriefings served a dual role, namely, providing methodological validity by aligning the wargame with real-world ATS dynamics and enabling its pedagogical value through collaborative learning among participants.

### Epistemological and Pedagogical Benefits

Two testing events confirmed that this manual/seminar wargaming can support aircraft product development, business and manufacturing planning. The benefits of using such a wargaming framework for product development and production strategy exploration are drawn from qualitative feedback collected from participants, highlighting both epistemological and pedagogical advantages.

The epistemological benefits of the wargaming exercises stemmed from helping to increase participants’ understanding of complex sociotechnical systems involving multiple stakeholders with disparate goals and objectives, underlying dynamics and their complex relationships. The testing succeeded in elaborating aspects of the ATS design problem that the participants had not considered or fully explored previously, such as the contrast between airline timescales (typically in the next few weeks/months) and the manufacturer timescales (typically over years or decades), which were experienced firsthand by the manufacturing team during the game. Moreover, it is noted that even in its proof-of-concept form, behaviours observed for real-life airlines were replicated by participants. One such example is the spontaneous emergence of the assembly line production slot auction process between competing airline teams and aircraft manufacturers.

The wargaming provides educational benefits by allowing participants to explore how air transport system stakeholders might respond to a given scenario. Through this experiential learning, participants develop strategic thinking and gain better understanding of decision-making on aircraft purchasing, route expansion, revenue generation, and investment strategies in FALs or aircraft development programmes. More specifically, the wargaming facilitates training on the behaviour, tactics, and dynamics observed between airlines, airports and manufacturers in the ATS. In addition, the game increased buy-in from participants, primarily from Airbus UK, in these two iterations. However, it potentially can be extended to a wider audience including customers and partners in the future as they have actively helped to the development of future products and production strategies. This will promote a sense of ownership and deeper understanding in the final envisaged/targeted context.

The wargame testing events reported in this paper took place in a single day. For educational purposes, a shorter session such as a one-day exercise can suffice and effectively provide awareness and learning experiences. However, fully realising the epistemological benefits requires more than a single day. A more extended exercise, such as a 3–5-day wargaming session is necessary for comprehensive scenario evaluation and deeper understanding. To be useful for guiding decision-making or developing strategy, it is also essential to conduct a plurality of games to explore variations of scenarios and sensitivity to factors within those scenarios. Investing time and resources in a longer session is crucial to thoroughly explore complex scenarios and gain the in-depth knowledge needed for informed decision-making and robust strategy development.

### Limitations

Although this study demonstrates the potential of the proposed wargaming framework in supporting aircraft manufacturers’ product and industrial strategy, some limitations are recognised. The validation of the framework was undertaken through two iterations of wargaming exercises, each conducted as a single-day workshop. These sessions effectively captured key interactions and yielded valuable insights, nevertheless, extended and repeated exercises would facilitate a more comprehensive exploration of complex, long-term dynamics within the ATS. Furthermore, the long gap between wargaming iterations led to different set of participants involved in each exercise. Future validations would benefit from repeated test with a closer gap between session and a consistent participant group to improve continuity, reliability of framework and reliability of findings. Moreover, the calibration of parameters (such as airlines’ initial financial resources and fleet composition) also presented challenges, with outcomes shown to be sensitive to these initial conditions, thereby indicating the need for further refinement and validation. In addition, the current version of the wargame remains largely manual in nature, requiring substantial facilitation effort and creating an inherent trade-off between realism and playability. Finally, the framework presently models only two competing airlines and a single manufacturer, without incorporating other critical stakeholders such as airports, regulators, or rival manufacturers, thus limiting the breadth of system interactions represented. These limitations delineate areas for further development aimed at enhancing the framework’s scalability, representativeness and applicability across a broader range of industrial contexts.

## Conclusion and Recommendation for Further Work

Although wargaming has been used in business contexts, it is not widely adopted, and its use in the area of ATS is particularly rare. Unlike traditional planning or analytical methods, wargaming is particularly suited to the modelling and simulation of airline competition and hierarchical interactions of airlines and manufacturers. This method enables participants to explore how airlines and manufacturers interact across multiple time horizons, revealing interdependencies and trade-offs that are difficult to capture through traditional analytical planning methods such as linear programming or regression-based forecasting. It provides a structured environment in which the dynamic, uncertain, and emergent nature of airline rivalry and aircraft development can be explored. It also serves as an experiential learning tool, helping and preparing decision-makers to understand the complexity and to develop adaptive strategies in response to evolving scenarios.

This article proposed a wargaming framework, comprising essential game elements including objectives, players, game components, game mechanics, scenarios, facilitation and debriefing sessions. It was designed to support manufacturers’ product development and industrial strategy by following rigorous stages of design, preparation, testing and evaluation. The framework development was facilitated through two collaborative development iterations between teams from Cranfield University and Airbus UK. By adapting military wargaming principles into ATS context, this framework enabled the participants to capture a holistic perspective on airline and aircraft manufacturer competitions including the social and human behavioural aspects of decision-making. The two design and validation iterations demonstrated that the combined game components, mechanics and scenarios provides a simplified yet highly representative view of airline operations. This allows players to perceive the real-world operational dynamics, such as the stark contrast between airline time scales (e.g., tomorrow or next week) and OEM time scales (e.g., developing aircraft over 5+ years). Moreover, the wargaming exercises revealed aspects of the ATS problem that participants had not previously considered, the emergent processes arising from participant interactions. For instance, the spontaneous creation of production slot auctions, which were not originally planned for.

Several avenues for future research and development of the proposed wargaming framework have been identified. Subsequent studies should extend the wargaming exercises beyond single-day workshops to longer and more iterative sessions, thereby enabling a more comprehensive examination of scenarios and a deeper understanding of the complex dynamics inherent within the ATS. Further iterations would also facilitate the calibration and validation of key parameters, such as airline financing, fleet size and production capacity, to improve representativeness and reliability of the outcomes. Expanding the framework to encompass additional stakeholders (including airports, regulatory bodies and competing manufacturers) would provide a more holistic representation of stakeholder interactions within the ATS. Further refinements are likewise required to optimise the balance between realism and playability, including enhanced automation of calculations and visualisations to reduce administrative effort and improve participant experience. In addition, future studies may explore a broader range of strategic themes relevant to aircraft manufacturers, such as market share and profit growth, the introduction of new technologies and aircraft capabilities, pricing and marketing strategies, the effects of technological or environmental disruptions, and the influence of policy and regulatory developments. Advancing the framework along these dimensions would strengthen its capacity to support evidence-based decision-making and strategic planning within complex and evolving industrial environments.
